# Regulation between personality traits: individual social tendencies modulate whether boldness and leadership are correlated

**DOI:** 10.1098/rspb.2018.0829

**Published:** 2018-06-13

**Authors:** Peggy A. Bevan, Isabella Gosetto, Eliza R. Jenkins, Isobel Barnes, Christos C. Ioannou

**Affiliations:** School of Biological Sciences, University of Bristol, Bristol, BS81TQ, UK

**Keywords:** personality, boldness, sociability, conformity, leadership, decision-making

## Abstract

Although consistent behavioural differences between individuals (i.e. personality variation) are now well established in animals, these differences are not always expressed when individuals interact in social groups. This can be key in important social dynamics such as leadership, which is often positively related to personality traits such as boldness. Individuals consistently differ in how social they are (their sociability), so if other axes of personality variation, such as boldness, can be suppressed during social interactions, this suppression should be stronger in more sociable individuals. We measured boldness (latency to leave a refuge when alone) and sociability (time spent with a conspecific) in three-spined sticklebacks (*Gasterosteus aculeatus*) and tested the boldness–leadership association in pairs of these fish. Both boldness and sociability were repeatable, but were not correlated. When splitting the data between the 50% most sociable and 50% less sociable fish, boldness was more strongly associated with leadership in less rather than more sociable individuals. This is consistent with more sociable fish conforming to their partner's behaviour due to their greater social tendency. One axis of personality variation (sociability) can thus modulate the relationship between others (boldness and leadership), with potential implications for selection on personality variation in social animals.

## 1. Introduction

Consistent inter-individual differences in behaviour within populations, referred to as personality variation, are now known to be a widespread phenomenon across animal taxa [1,2]. Boldness is one such axis of personality variation, i.e. a personality trait, and describes consistent differences between individuals in their response to perceived risk [1]. Boldness is generally considered to be part of a major ‘proactive–reactive’ axis of personality variation, where boldness is one of a suite of behaviours including exploration, activity and aggression that correlate positively with one another [3]. Variation in boldness is believed to result from differences in a growth–mortality trade-off [4], driven by bolder individuals having greater food intake when foraging [5,6] but a higher risk from predation [7]. These factors of foraging and risk are also major determinants in whether individuals are leaders in groups. Leadership in animals occurs when a single or small minority of individuals disproportionally influence group decisions such as when and where to initiate behaviours [8]. With greater influence, leaders can determine group behaviour by directing others to resources when the leaders are in greater need [9], and in groups that are led from the front, leaders have greater access to encountered food [8]. However, leadership can be costly and increase a leader's risk of predation [8], possibly because their attention to other tasks while navigating is compromised [10]. The parallel between boldness and leadership in the foraging–risk trade-off is consistent with bolder individuals having a greater tendency to lead, with boldness and leadership often being positively correlated [11,12].

Personality variation, and more overt sources of consistent variability between individuals such as age and sex, can conflict with living in groups [5]. The maintenance of group cohesion often requires individuals to synchronize their behaviour with others, so that some or all group members may not always be able to express their preferred behaviour when these preferences differ [13]. This conflict can result in individuals conforming to one another [14], with behaviour in groups being determined by the most or least bold, active or proactive individual. A number of studies have explored if personality variation is expressed in social groups and have shown mixed results. Studies of risk-taking behaviour in perch [15], sticklebacks [16], mud crabs [17] and nutmeg mannikins [18] show correlations in individual behaviour between asocial and social contexts. However, when performing a risky behaviour such as crossing an exposed area from a refuge to a food source, consensus decisions within the group are more likely, resulting in conformity that suppresses personality variation being expressed in groups [5]. This effect suggests that personality variation will be less evident within groups of more sociable animals, as the greater tendency for social contact is expected to result in greater conformity.

In addition to variation in the tendency to be social between populations [19], variability in social tendency has also been demonstrated to be consistent between individuals within populations. Studies demonstrating this sociability as an axis of personality variation often measure the time a focal individual spends near to stimulus conspecifics [6,20]. While extensive research has examined how different axes of personality variation are related to one another (i.e. behavioural syndromes [3,21]), it has yet to be shown that one axis of personality variation can influence whether others are correlated. Sociability would be a strong candidate to have such a modulating effect: more sociable individuals will value group cohesion more highly [22], resulting in them being more likely to conform to other group members' behaviour and suppressing the expression of other aspects of their personality [5]. This mechanism suggests that the behaviour of more sociable individuals is less predictable from their other personality traits when in a social context.

Here, we test whether the often observed correlation between boldness and leadership during group decision-making is affected by individual social tendencies, using three-spined sticklebacks (*Gasterosteus aculeatus*) as a model system. Relatively bolder sticklebacks tend to initiate movements compared to their shyer group mates and hence take the role of leader if they are followed [16,23,24]. Additionally, once a shoal is on the move, bolder individuals have been found to be more likely to occupy frontal positions [5]. In sticklebacks, leadership can thus be considered part of a proactive–reactive behavioural syndrome, but the positive relationship between leadership and boldness may weaken in more sociable individuals. On each day for two consecutive days, three-spined sticklebacks were tested for their sociability, tested alone in a Y maze and later retested in the Y maze in pairs, i.e. each test took place twice. Boldness when tested alone was used to predict behaviour of individuals in pairs when tested on the other day of testing. We hypothesized that for less sociable fish, individual boldness would influence leadership in pairs, while it would be a weaker, or not a statistically significant, predictor of leadership in more sociable fish.

## 2. Material and methods

### (a) Subjects and housing

**Figure 1.**
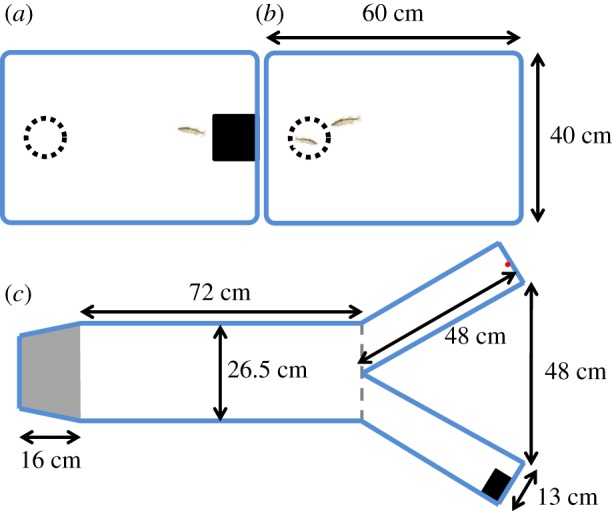
Overhead view of the holding tank used to house individual fish alone (*a*), the holding tank when adapted for the sociality tests (*b*) and the Y maze used for the individual boldness assay and paired fish trials (*c*). Each holding tank held a single fish, with water depth of 9 cm. The refuge (consisting of two square walls and roof made of black plastic, 10 × 10 × 8 cm (width × length × height)) was provided for the fish while housed, but was removed during the sociability trials (*b*). The clear plastic cup (dotted circles, 500 ml: 9 cm diameter at the top, 6 cm at the bottom and 12 cm high, containing 2 cm of aquarium gravel) was present throughout and held the companion individual in the sociability trials. Pilot trials were carried out and demonstrated that the fish spent substantially more time near the cup with gravel when it contained a conspecific than when it was empty. These holding tanks were arranged into two groups of four, so that a single camera mounted above four tanks recorded four trials at once. White plastic sheeting was placed underneath the clear plastic tanks to produce a white background to filming from above. Tanks were visually isolated from one another and outside disturbance by opaque white partitions. The Y maze was used to assess boldness (refuge use) of individual fish, and later the same day, behaviour in pairs of fish. Fish were habituated in the refuge (grey-shaded area) before the door was raised allowing access into the rest of the tank. At the end of each arm was either a food stimulus (red circle) or small refuge (black rectangle). Each figure is approximately to scale. (Online version in colour.)

Three-spined sticklebacks (mean length ± s.d. = 4.50 ± 0.62 cm) were caught from the River Cary, Somerset, UK (grid ref: ST 469 303) and housed in glass holding tanks (40 × 70 × 34 cm (width × length × height)) on a flow-through system in a temperature-controlled room (15–16°C) at the University of Bristol. Fish were kept on a constant light regime (12 L : 12 D cycle) and fed on defrosted bloodworms daily. Ten sets of eight fish (*n* = 80 fish total) were tested between 17 October and 25 November 2016. Each test fish in a set was individually moved to its own plastic tank the afternoon before testing began the next day where it was held alone for the duration of the experiment (see figure 1 for details). At the end of each day, all individuals were fed three to four bloodworms. Fish were not re-used after testing and were returned to separate glass holding tanks for use in later experiments. The standard body length (mm) of each fish was measured from the videos of the Y maze trials.

### (b) Experimental procedure: sociability tests

The morning (between 10:00 and 11:00) after the day fish were transferred to their holding tanks and on the day following this, sociability tests were carried out in the individual holding tanks. A randomly selected conspecific was netted from non-experimental holding tanks into a plastic cup in the holding tank, the refuge was removed (figure 1) and filming began. A single conspecific was used as a stimulus as the group decision-making trials used pairs of fish (thus, each fish only had a single partner), with a different stimulus conspecific being used for each of the eight test fish in the sociability tests. Behaviour of the test fish was recorded using GoPro Hero5 cameras filming from above. The tanks were filmed for a total of 900 s. In the subsequent video analysis, the first 300 s were not analysed to allow the fish to habituate from any disturbance. In the following 600 s, the number of seconds each fish spent within one body length of the cup containing the companion fish was recorded. The plastic cup did not allow physical or olfactory interaction between the companion fish and the test fish, while still allowing visual cues. Fish were not used as test subjects if they had already been used as a companion fish.

### (c) Experimental procedure: Y maze trials

In the afternoons (between 13:00 and 16:00) on both days following the sociability tests, each fish was tested alone and then in a pair in a Y maze to assess decision-making in asocial and social contexts. Trials were carried out in a white Perspex Y-shaped arena, filled to a depth of 11 cm (figure 1). Water was kept aerated and filtered when fish were not being tested in the arena. Trials were filmed from above using a remotely controlled Panasonic HDC-SD800 video camera at a resolution of 1920 × 1080 and frame rate 25 f s^−1^, held on a tripod 125 cm above the arena. The arena was surrounded by white sheeting and the camera was connected to a monitor, so the fish could be observed without disturbance. A refuge at the base of the Y was sectioned off by a door that could be raised by a remote pulley. At the other end of the tank in the arms of the Y, we presented two different stimuli to the fish (food and refuge, one in each arm) to differentiate the two arms more than if the arms were empty or contained the same stimuli. The refuge consisted of a small piece of black corrugated plastic (5 × 7 cm) at the water surface and the food stimulus was a plastic pipette with red electrical tape wrapped (8 × 3 mm (length × diameter)) around the tip, attached vertically to the wall of the arena so the red tape was just beneath the surface of the water and clearly visible to the fish. This acted as a food stimulus [23] as it is similar to the bloodworm fed to the fish, and red on a white background is known to be highly conspicuous to three-spined sticklebacks [25]. It was used in favour of real bloodworm as it is easily standardized and prevents the fish responding to olfactory cues. Which of the two stimuli were in the left or right arm of the maze was randomized for each trial. The shape of the arena and positioning of the stimuli were designed so that the fish were unable to see the stimuli from inside or in front of the refuge and instead had to swim into the open stem of the Y.

Within the asocial and then social tests, fish (or pairs) were tested in a randomized order. Fish were first tested alone. Individual fish were moved from their holding tank and placed in the darkened (with 5 mm black plastic mesh overhead) refuge at the start of the maze and given 120 s to habituate before recording began and the door was slowly raised, giving the fish access to the main arena (figure 1). The latency for a fish to first leave the refuge at the start of the maze (to the nearest second) was recorded as a measure of refuge use and hence boldness. A decision was defined as being made when the fish's midpoint first crossed the decision line at the junction in the Y, with the fish swimming into an arm of the maze (dashed line in figure 1). The latency to leave the refuge was recorded when the fish first spent more than 10 s outside of the refuge [20]. As the fish frequently swam directly to the stimulus at the end of the arms, this threshold was not applied to the decision of which arm was chosen. After all single-fish tests were completed that day, paired trials were conducted where the procedure was repeated for two experimental fish from the holding tanks tested together (both of which had been tested earlier that day in both sociability and asocial Y maze trials). The pairs were selected by size so each pair had a notable difference between a larger and smaller fish, allowing individual identities to be tracked without tagging with markers (body size was factored into the statistical analyses). The same pairs were tested on both days. In the paired trials, in addition to recording the time taken to first leave the refuge and first make a decision to swim into an arm, we recorded the identity of the fish that performed these initiating behaviours.

If a fish (or both fish in a pair) did not leave the refuge after 300 s, the trial was ended and the fish were given a maximum value of 300 s (there were missing data for the latency to choose an arm in these cases; see electronic supplementary material, table S1). If the fish left the refuge for more than 10 s, but did not choose an arm within 600 s from the refuge being left, the trial was also ended and latency to choose an arm was given a maximum value of 600 s.

### (d) Statistical analyses

The initial analyses focused on the single-fish trials and tested for repeatability within, and correlation between, boldness (latency to first leave the refuge in the Y maze when tested alone) and sociability (the time spent with a conspecific) to establish these as personality traits and to test whether they were independent variables. Spearman's rank correlation was used to test these relationships. To test whether the change in refuge use (i.e. consistency, the absolute difference in the latency to first leave the refuge on the first and second days of testing alone) varied significantly with whether individuals were classified as more or less sociable (see below), a generalized linear model (GLM) with a quasipoission error distribution (the data were overdispersed) was used. Spearman's rank correlation was also used to test whether this consistency measure correlated with the mean of each fish's two sociability scores. Establishing that consistency in boldness is unrelated to sociability is important in avoiding a potential confounding effect. If, for example, more sociable individuals are less consistent, then a reduced correlation between boldness and leadership may be because boldness is more variable over time in more sociable fish. Instead, we were interested in whether the social context of the paired fish trials affects the boldness–leadership relationship differently depending on individuals' sociability.

To investigate the relationship between boldness and leadership in the paired fish trials, generalized linear mixed models (GLMMs) were used, summarized in tables 1–4. Four response variables were analysed as measures representing leadership: whether an individual initiated leaving the refuge (binomially distributed), the time taken to leave the refuge by this initiating fish (negatively binomially distributed); whether an individual initiated into an arm of the maze (binomially distributed), the time taken to enter the arm by this fish (negatively binomially distributed). Each response variable was analysed separately and as a function of an individual's boldness (log10 transformed) and body length as covariates (presented in the main text; electronic supplementary material, table S2 shows the analysis with sociability also included). Each response variable was analysed using three models for each. In the first, all trials were included where that event occurred (e.g. a fish left the refuge). The data were then split depending on whether each fish was more or less sociable than the median sociability in that data, and the models rerun on each of these split datasets. In other words, the sociability categorization was determined by whether each fish's mean sociability (i.e. mean time spent with a conspecific) was greater (more sociable) or less (less sociable) than the median mean sociability of fish included in that analysis (electronic supplementary material, table S1). This approach to testing for the effect of sociability on the boldness–leadership relationship was used in favour of including a sociability × boldness term as an interaction would only test whether the slope between boldness and leadership differed with sociability (see electronic supplementary material, table S2, for details and results using this interaction approach). Instead, our hypothesis is to test whether the effect of boldness is more difficult to detect in more sociable individuals; for example, the slopes may be the same for more sociable and less sociable fish, but there may be more variation around the slope in the more sociable fish.

**Table 1.** Results of models explaining variance in which fish in each pair initiated leaving the refuge. Each row shows the result from a different model, which differ based on the explanatory variables included and/or the individuals included in the data analysed. The first set of rows are from the models with all trials included where a fish left the refuge, and the following sets are from the models where these data are split by whether fish are more or less sociable. SBL is the standard body length and the null model is the model lacking any explanatory variables. d.f. refers to degrees of freedom and ΔAICc refers to the difference in the corrected Akaike Information Criterion between the model and the most likely model. Models are ordered within each of the differing datasets by increasing ΔAICc.

whether individual initiates leaving the refuge			
sample	model	d.f.	ΔAICc
132	boldness	4	0.0
	boldness + SBL	5	1.9
	null	3	4.7
	SBL	4	5.2
65 (more sociable fish)	null	3	0.0
	boldness	4	0.3
	SBL	4	2.2
	boldness + SBL	5	2.6
65 (less sociable fish)	boldness	4	0.0
	boldness + SBL	5	1.8
	null	3	2.7
	SBL	4	3.1

**Table 2.** Results of models explaining variance in the time taken for a fish to first leave the refuge. See table 1 legend for details.

time taken to leave the refuge by the initiating fish			
sample	model	d.f.	ΔAICc
66	boldness	4	0.0
	boldness + SBL	5	0.9
	null	3	5.3
	SBL	4	7.6
33 (more sociable fish)	boldness	4	0.0
	null	3	2.1
	boldness + SBL	5	2.8
	SBL	4	4.5
33 (less sociable fish)	boldness	4	0.0
	boldness + SBL	5	2.1
	null	3	2.9
	SBL	4	5.4

**Table 3.** Results of models explaining variance in which fish in each pair initiated the first movement into an arm of the maze. See table 1 legend for details.

whether individual initiates into an arm of the maze			
sample	model	d.f.	ΔAICc
116	boldness	4	0.0
	boldness + SBL	5	0.4
	null	3	1.7
	SBL	4	3.4
58 (more sociable fish)	null	3	0.0
	boldness	4	2.2
	SBL	4	2.2
	boldness + SBL	5	4.5
58 (less sociable fish)	boldness	4	0.0
	boldness + SBL	5	0.1
	null	3	4.2
	SBL	4	6.0

**Table 4.** Results of models explaining variance in the time taken for a fish to first enter an arm of the maze. See table 1 legend for details.

time taken to enter arm by the initiating fish			
sample	model	d.f.	ΔAICc
58	boldness + SBL	5	0.0
	SBL	4	7.7
	boldness	4	14.6
	null	3	15.8
29 (more sociable fish)	null	3	0.0
	SBL	4	0.2
	boldness	4	2.6
	boldness + SBL	5	3.0
29 (less sociable fish)	boldness + SBL	5	0.0
	SBL	4	11.9
	boldness	4	12.4
	null	3	14.4

To determine the importance of boldness and body length in whether an individual was the initiator and the time taken to initiate, models with and without each covariate were compared using the difference in the corrected Akaike Information Criterion between the model and the most likely model (the ΔAICc). Models with lower AIC are more likely given the data, with the most likely model having a ΔAICc of zero. Support for other models can be considered strong if their AICc is within two units of the most likely model [26], although more parsimonious models (those with fewer parameters) should be favoured given similar AICc values. By comparing the covariates present in the most likely and strongly supported models, it can be inferred which covariates are important to include in explaining the variance in the response variable. Results based on *p*-values are also given in electronic supplementary material, table S1, and broadly agree with those from this AIC approach.

All analyses of paired fish tests included pair identity as a random intercept (electronic supplementary material, table S1). In tests which included data from both fish in a paired trial (for example, whether a fish was an initiator or not as a function of their boldness), trial identity was nested in pair identity as the random effect (electronic supplementary material, table S1). Including these random effects accounted for the non-independence in the data, where data from different fish in the same trial or the same pair in multiple trials were included. Full details of each GLMM are given in electronic supplementary material, table S1, including results for the effect of body length. The dispersion parameters for the GLMMs were checked to be approximately equal to one, i.e. between 0.5 and 2. All analyses were carried out using R v. 3.3.3.

## 3. Results

### (a) Single-fish tests

Analysis of the single-fish trials revealed that both boldness and sociability were repeatable and hence are stable over a short period of time. The two variables were not correlated with one another, and the change in boldness between the two trials (i.e. their consistency) was not related to individuals' sociability (electronic supplementary material, figures S1 and S2). There was no evidence that boldness was more repeatable in less sociable fish (electronic supplementary material, figure S3). Larger fish were found to be less bold, but body length did not correlate with sociability or consistency in boldness (electronic supplementary material, figure S1).

### (b) Paired fish tests: initiating leaving the refuge

**Figure 2.**
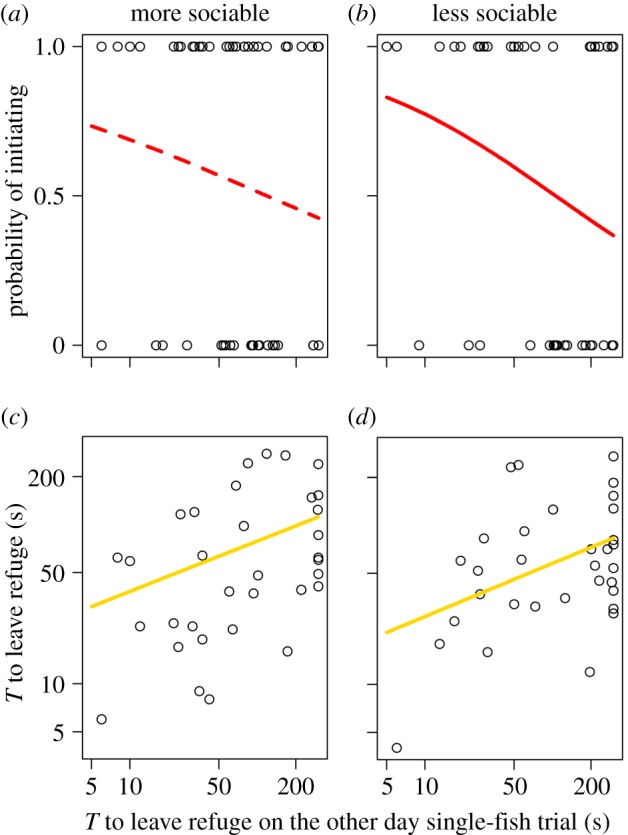
The effect of individual boldness on whether a fish initiated the first movement from the refuge (*a*,*b*) and the time taken to do so (*c*,*d*) in paired fish tests. Boldness is measured as the time taken to leave the refuge when tested alone on the other day (shown on a log10 scale), where smaller values indicate bolder fish. Individuals are split between those that are more (*a*,*c*) and less (*b*,*d*) sociable in each analysis. The lines show the fitted relationships from the GLMMs. Solid and dashed lines indicate significant (*p* < 0.05) and non-significant (*p* > 0.05) effects, respectively (electronic supplementary material, table S1). (Online version in colour.)

To analyse leadership in pairs of fish, models considered different response variables with an individual's boldness as a predictor and body length also included as a covariate, initially with all data included, and then tests were repeated for the most and least sociable fish separately. Boldness (log10 time to leave refuge when tested alone in the Y maze on the other day) determined which fish in the pair initiated leaving the refuge (models including boldness as a covariate were supported based on the ΔAICc, table 1), demonstrating a positive correlation between boldness and leadership behaviour (figure 2*a*,*b*). When repeating this analysis for the more and less sociable fish separately, the effect of boldness remained for the less sociable fish (figure 2*b* and table 1; slope: −0.52, s.e. of slope: 0.25), but including boldness did not improve the model fit compared to the null model for the more sociable fish (figure 2*a* and table 1; slope: −0.32, s.e. of slope: 0.23). This suggests that the relationship between boldness and leadership was reduced in more sociable individuals.

The time taken to leave the refuge by the initiating fish, rather than who initiated, was longer in less bold initiators (table 2), again showing a positive correlation between boldness and leadership behaviour (figure 2*c*,*d*). This effect remained for both more (figure 2*c*; slope: 0.32, s.e. of slope: 0.14) and less (figure 2*d*; slope: 0.33, s.e. of slope: 0.14) sociable initiators, with the slopes of the relationship and their variabilities being similar. In all cases, models including boldness were more likely than those lacking this covariate (table 2).

### (c) Paired fish tests: decision to enter an arm of the maze

**Figure 3.**
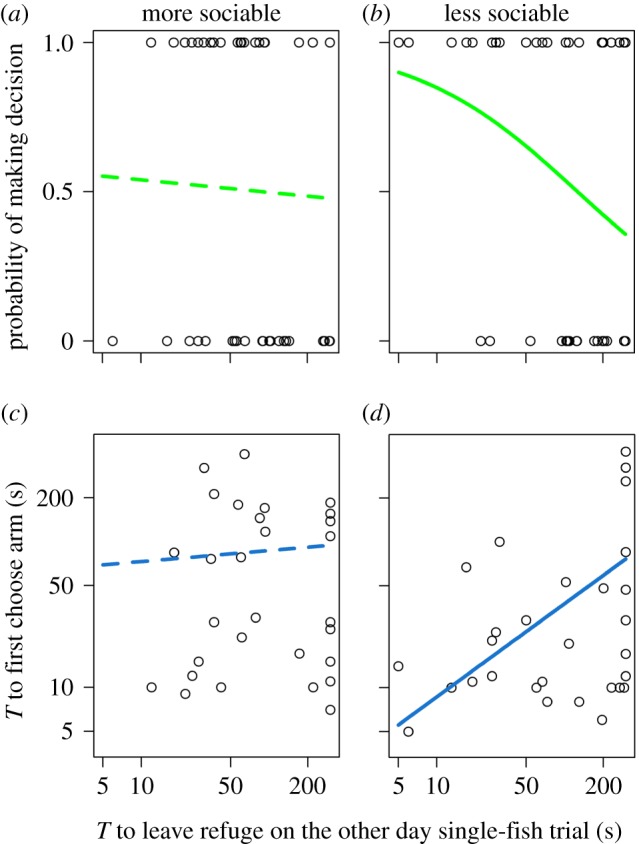
The effect of individual boldness on whether a fish initiated the first movement into an arm of the Y maze (*a*,*b*) and the time taken to do so (*c*,*d*) in paired fish tests. Boldness is measured as the time taken to leave the refuge when tested alone on the other day (shown on a log10 scale), where smaller values indicate bolder fish. Individuals are split between those that are more (*a*,*c*) and less (*b*,*d*) sociable in each analysis. The lines show the fitted relationships from the GLMMs; in (*c*) and (*d*), these fitted lines include the main effect of fish body length fitted at the mean value for body length as this variable was significant in less sociable fish. Solid and dashed lines indicate significant (*p* < 0.05) and non-significant (*p* > 0.05) effects, respectively (electronic supplementary material, table S1). (Online version in colour.)

The bolder fish in a pair tended to be more likely to first choose an arm of the maze and make the first decision, although the null model was also supported from the ΔAICc (table 3; the effect of boldness was only just statistically significant, see electronic supplementary material, table S1). A reason for this lack of a strong effect of boldness on this leadership behaviour is that boldness was not a predictor of which fish initiated the first movement into an arm in the more sociable fish (figure 3*a* and table 3; slope: −0.073, s.e. of slope: 0.24). By contrast, in less sociable fish, models including boldness were more likely than those without this covariate (table 3). Bolder individuals were significantly more likely to be the first fish to choose an arm (figure 3*b*; slope: −0.68, s.e. of slope: 0.29), again showing a stronger relationship between boldness and leadership in less sociable compared to more sociable fish.

Across all fish that initiated a movement into an arm of the Y maze, the most likely model describing the time taken to enter the arm included both boldness and standard body length as covariates (table 4). Initiators were faster to enter the arm if they were bolder (figure 3*c*,*d*), supporting a positive relationship between boldness and leadership, and smaller initiators took longer than larger fish. There was no evidence that boldness affected this latency in more sociable fish (figure 3*c* and table 4; slope: 0.076, s.e. of slope: 0.25). By contrast, the most likely model in the less sociable fish included boldness as a covariate (figure 3*d* and table 4; slope: 0.64, s.e. of slope: 0.12). Interestingly, whether fish were classed as more or less sociable also influenced the relationship between body length and the latency to initiate movement into an arm. As with the effect of boldness, there was weak evidence that body length was important in the model for the more sociable fish (the null model was the most likely, table 4; also see electronic supplementary material, table S1), while the most likely model for the less sociable fish included body length as well as boldness (the other models were poorly supported by the data, table 4).

## 4. Discussion

We quantified social tendency (sociability, the time spent with a stimulus conspecific) and risk-taking tendency (boldness, the latency to leave a refuge) in individuals and tested whether the positive relationship between boldness and leadership was affected by social tendency. In a social context when fish were tested in pairs, an individual's sociability modulated whether boldness was related to tendencies to lead, measured as the likelihood an individual would initiate movement from the refuge and, analysed separately, initiate movement into an arm of the maze. Consistent with our hypothesis, there was a weaker, or absent, relationship between boldness and leadership in more sociable individuals. Additionally, boldness was only a significant predictor of the latency to first enter an arm of the maze in less sociable individuals, and the effect of body length on this variable was also only present in less sociable individuals. These results demonstrate that the ability to detect the influence of boldness (and potentially, body length) on behaviour in groups was weaker in more sociable individuals. From an experimental perspective, this dependency on individuals' sociability suggests that factors which affect the degree of sociability will have an indirect effect on whether other behaviours are found to correlate when individuals are alone or in groups. For example, methods of catching individuals from the wild may be biased towards groups [27], or social tendencies may increase when perceived risk in the testing apparatus is higher [5]; in such cases, it is less likely that axes of personality variation such as boldness will correlate with behaviour in groups.

Boldness and sociability were not found to be correlated, indicating that these behaviours that are repeatable at the individual level are not part of the same behavioural syndrome [3,21]. This independence between sociability and boldness is important as the social trials could be viewed as a changed environment, and it has been shown previously that more proactive (i.e. bold) individuals are less flexible and responsive to changed conditions, instead developing rigid routines when environments are stable [28]. With sociability being uncorrelated to boldness, the greater predictive power of boldness in paired trials with less sociable fish cannot be explained by less sociable fish being less adaptable in general to a changed (either socially or otherwise) environment. Similarly, another possible explanation for the effect of sociability on whether boldness was important in a social context is that more sociable individuals are less consistent in their boldness. A lower repeatability of boldness in more sociable individuals would be expected to carry over to social contexts, and hence, boldness would be less influential in group trials because it is less consistent, and hence less influential, more generally. It has recently been established that there is inter-individual variation in how consistent individuals are in their behaviour, as well as the average levels of behaviour expressed [29]. We found no evidence, however, that the absolute difference in latencies to leave the refuge on different days when tested alone (a basic measure of consistency [23]) was related to the time spent with a conspecific (electronic supplementary material, figure S2). Moreover, the correlation between the latencies to leave the refuge when tested alone on different days was stronger, not weaker, in the fish classed as more rather than less sociable (electronic supplementary material, figure S3).

Although we used the minimum time between tests to check for repeatability, with tests occurring on consecutive days, our previous studies on personality variation in three-spined sticklebacks from the same population tested under similar conditions have demonstrated refuge use to be repeatable over longer time scales. Ioannou & Dall [23] found that the latency to first leave a refuge showed an overall correlation coefficient (*r*_s_) of 0.71, with 2–6 days between tests of the same individuals, and McDonald *et al*. [5] found that the same variable was repeatable (*r*_s_ = 0.37) with 3 days between tests. To our knowledge, the repeatability of individuals’ sociability has yet to be tested over longer time scales in this population, but previous work using other populations of this species have also shown sociability to be repeatable over longer periods of time [6,30]. We thus think it is likely that the repeatabilities found here are representative of personality variation over longer time scales, and hence unlikely that this could affect the overall conclusions of the study. If the repeatability scores are to be used for further study, such as in a meta-analysis [2], it should be considered, however, that the time between tests was only approximately 24 h.

Consistent inter-individual variation in behaviours such as boldness have ecological and evolutionary consequences [31,32]. Whether boldness when individuals are tested alone is expressed in groups will thus determine the influence of boldness on these processes in social animals [5]. The most direct negative consequence of greater boldness is likely to be the increased risk of mortality or injury [7]. In social groups, bolder individuals typically lead by initiating movements into riskier areas [11,24] and this can increase predation risk when groups are led from the front [8]. Alternatively, initiating individuals can fail to lead others, and become isolated [33], so that bolder individuals lose the safety gained from being in a group. However, bolder individuals often have greater access to food in social contexts [5,6,31]. Our results suggest that in populations of more sociable individuals, individual boldness is less likely to determine the tendency to initiate movements and hence be less important in determining the risk of predation or the intake of food. As social behaviour varies with ecological factors, including predation risk [19,34], there may be an interaction occurring where ecological factors influence average levels of sociability and boldness, sociability influences whether individual variation in boldness is expressed and hence whether boldness has an impact on ecological processes.

## Supplementary Material

Supplementary Results, Tables and Figures

## Supplementary Material

Data S1

## Data accessibility

The data from experiment for paper.csv file, included as supplementary, is the data required to recreate the analyses in the paper, and is freely available and accessible.

## References

[1] Réale D, Reader SM, Sol D, McDougall PT, Dingemanse NJ 2007 Integrating animal temperament within ecology and evolution. Biol. Rev. 82, 291–318. (10.1111/j.1469-185X.2007.00010.x) 17437562

[2] Bell AM, Hankison SJ, Laskowski KL 2009 The repeatability of behaviour: a meta-analysis. Anim. Behav. 77, 771–783. (10.1016/j.anbehav.2008.12.022) 24707058 PMC3972767

[3] Sih A, Bell A, Johnson JC 2004 Behavioral syndromes: an ecological and evolutionary overview. Trends Ecol. Evol. 19, 372–378. (10.1016/j.tree.2004.04.009) 16701288

[4] Stamps JA 2007 Growth-mortality tradeoffs and ‘personality traits’ in animals. Ecol. Lett. 10, 355–363. (10.1111/j.1461-0248.2007.01034.x) 17498134

[5] McDonald ND, Rands SA, Hill F, Elder C, Ioannou CC 2016 Consensus and experience trump leadership, suppressing individual personality during social foraging. Sci. Adv. 2, e1600892 (10.1126/sciadv.1600892) 27652342 PMC5023318

[6] Ward AJW, Thomas P, Hart PJB, Krause J 2004 Correlates of boldness in three-spined sticklebacks (*Gasterosteus aculeatus*). Behav. Ecol. Sociobiol. 55, 561–568. (10.1007/s00265-003-0751-8)

[7] Bell AM, Sih A 2007 Exposure to predation generates personality in threespined sticklebacks (*Gasterosteus aculeatus*). Ecol. Lett. 10, 828–834. (10.1111/j.1461-0248.2007.01081.x) 17663716

[8] Krause J, Hoare D, Krause S, Hemelrijk CK, Rubenstein DI 2000 Leadership in fish shoals. Fish Fish. 1, 82–89. (10.1111/j.1467-2979.2000.tb00001.x)

[9] Webster MM 2016 Experience and motivation shape leader–follower interactions in fish shoals. Behav. Ecol. 28, 77–84. (10.1093/beheco/arw133)

[10] Piyapong C, Morrell LJ, Croft DP, Dyer JRG, Ioannou CC, Krause J 2007 A cost of leadership in human groups. Ethology 113, 821–824. (10.1111/j.1439-0310.2007.01382.x)

[11] Beauchamp G 2000 Individual differences in activity and exploration influence leadership in pairs of forading zebra finches. Behaviour 137, 301–314. (10.1163/156853900502097)

[12] Aplin LM, Farine DR, Mann RP, Sheldon BC 2014 Individual-level personality influences social foraging and collective behaviour in wild birds. Proc. R. Soc. B 281, 20141016 (10.1098/rspb.2014.1016) PMC4100518 24990682

[13] Fürtbauer I, Fry A 2018 Social conformity in solitary crabs, *Carcinus maenas*, is driven by individual differences in behavioural plasticity. Anim. Behav. 135, 131–137. (10.1016/j.anbehav.2017.11.010)

[14] Brown C, Laland KN 2002 Social learning of a novel avoidance task in the guppy: conformity and social release. Anim. Behav. 64, 41–47. (10.1006/anbe.2002.3021)

[15] Magnhagen C, Bunnefeld N 2009 Express your personality or go along with the group: what determines the behaviour of shoaling perch? Proc. R. Soc. B 276, 3369–3375. (10.1098/rspb.2009.0851) PMC2817179 19586948

[16] Harcourt JL, Ang TZ, Sweetman G, Johnstone RA, Manica A 2009 Social feedback and the emergence of leaders and followers. Curr. Biol. 19, 248–252. (10.1016/j.cub.2008.12.051) 19185497

[17] Belgrad BA, Griffen BD 2017 Habitat quality mediates personality through differences in social context. Oecologia 184, 431–440. (10.1007/s00442-017-3886-4) 28528392

[18] Rieucau G, Morand-Ferron J, Giraldeau L-A 2010 Group size effect in nutmeg mannikin: between-individuals behavioral differences but same plasticity. Behav. Ecol. 21, 684–689. (10.1093/beheco/arq039)

[19] Herbert-Read JE et al 2017 How predation shapes the social interaction rules of shoaling fish. Proc. R. Soc. B 284, 20171126 (10.1098/rspb.2017.1126) PMC5577484 28855361

[20] Cote J, Fogarty S, Weinersmith K, Brodin T, Sih A 2010 Personality traits and dispersal tendency in the invasive mosquitofish (*Gambusia affinis*). Proc. R. Soc. B 277, 1571–1579. (10.1098/rspb.2009.2128) PMC2871838 20071380

[21] Sih A, Cote J, Evans M, Fogarty S, Pruitt J 2012 Ecological implications of behavioural syndromes. Ecol. Lett. 15, 278–289. (10.1111/j.1461-0248.2011.01731.x) 22239107

[22] Trompf L, Brown C 2014 Personality affects learning and trade-offs between private and social information in guppies, *Poecilia reticulata*. Anim. Behav. 88, 99–106. (10.1016/j.anbehav.2013.11.022)

[23] Ioannou CC, Dall SRX 2016 Individuals that are consistent in risk-taking benefit during collective foraging. Sci. Rep. 6, 33991 (10.1038/srep33991) 27671145 PMC5037426

[24] Nakayama S, Harcourt JL, Johnstone RA, Manica A 2016 Who directs group movement? Leader effort versus follower preference in stickleback fish of different personality. Biol. Lett. 12, 20160207 (10.1098/rsbl.2016.0207) 27194292 PMC4892248

[25] Ioannou CC, Krause J 2009 Interactions between background matching and motion during visual detection can explain why cryptic animals keep still. Biol. Lett. 5, 191–193. (10.1098/rsbl.2008.0758) 19158025 PMC2665835

[26] Burnham KP, Anderson DR 2002 Model selection and multimodel inference: a practical information-theoretic approach. New York, NY: Springer-Verlag.

[27] Rieucau G, Fernö A, Ioannou CC, Handegard NO 2015 Towards of a firmer explanation of large shoal formation, maintenance and collective reactions in marine fish. Rev. Fish Biol. Fish. 25, 21–37. (10.1007/s11160-014-9367-5)

[28] Koolhaas JM, de Boer SF, Coppens CM, Buwalda B 2010 Neuroendocrinology of coping styles: towards understanding the biology of individual variation. Front. Neuroendocrinol. 31, 307–321. (10.1016/j.yfrne.2010.04.001) 20382177

[29] Biro PA, Adriaenssens B 2013 Predictability as a personality trait: consistent differences in intraindividual behavioral variation. Am. Nat. 182, 621–629. (10.1086/673213) 24107369

[30] Jolles JW, Boogert NJ, Sridhar VH, Couzin ID, Manica A 2017 Consistent individual differences drive collective behavior and group functioning of schooling fish. Curr. Biol. 27, 2862–2868. (10.1016/j.cub.2017.08.004) 28889975 PMC5628957

[31] Ioannou CC, Payne M, Krause J 2008 Ecological consequences of the bold-shy continuum: the effect of predator boldness on prey risk. Oecologia 157, 177–182. (10.1007/s00442-008-1058-2) 18481092

[32] Dall SRX, Bell AM, Bolnick DI, Ratnieks FLW 2012 An evolutionary ecology of individual differences. Ecol. Lett. 15, 1189–1198. (10.1111/j.1461-0248.2012.01846.x) 22897772 PMC3962499

[33] Ioannou CC, Singh M, Couzin ID 2015 Potential leaders trade off goal-oriented and socially-oriented behavior in mobile animal groups. Am. Nat. 186, 284–293. (10.1086/681988) 26655156

[34] Ioannou CC, Ramnarine IW, Torney CJ 2017 High-predation habitats affect the social dynamics of collective exploration in a shoaling fish. Sci. Adv. 3, e1602682 (10.1126/sciadv.1602682) 28508069 PMC5415332

