# Supplementary material for: Regulation between personality traits: individual social tendencies modulate whether boldness and leadership are correlated
Source: Proc Biol Sci. 2018 Jun 13;285(1880):20180829. doi: 10.1098/rspb.2018.0829 (PMC6015863; doi:10.1098/rspb.2018.0829)
Supplement: Supplementary Results, Tables and Figures [file rspb20180829supp1.docx]

Regulation between personality traits: Individual social tendencies modulate whether boldness and leadership are correlated

Supplementary material

Published in Proceedings of the Royal Society B.

Peggy A. Bevan, Isabella Gosetto, Eliza R. Jenkins, Isobel Barnes, Christos C. Ioannou

School of Biological Sciences, University of Bristol, Bristol, BS81TQ

Supplementary text: Results from single-fish tests

The latency to leave the refuge was positively correlated in tests on different days for three-spined sticklebacks when tested alone (figure S1A; Spearman’s rank correlation: n = 80, r_s_ = 0.39, p < 0.001). There was also a high degree of correlation in sociability on different days, measured as the time spent with a stimulus conspecific in the absence of a refuge (figure S1B; n = 80, r_s_ = 0.69, p < 0.001). The latency to leave the refuge (boldness) and sociability were not significantly correlated with one another, even though these measures were compared within each day, i.e. the time spent with a conspecific was not correlated with the latency to leave the refuge later that same day (figure S1C; n = 160, r_s_ = -0.11, p = 0.15). Body length correlated positively with the latency to leave the refuge (mean over the two trials; figure S1D; Spearman’s rank: n = 80, r_s_ = 0.34, p = 0.0020). Body length did not correlate significantly with sociability (mean over the two trials; figure S1E; Spearman’s rank: n = 80, r_s_ = -0.11, p = 0.33).

To test whether greater sociability was associated with a reduced consistency in boldness, consistency was measured as the absolute change in time to leave the refuge in the two Y maze tests (i.e. boldness on days 1 and 2). There was no correlation between sociability and consistency in boldness (figure S2A; Spearman’s rank correlation: n = 80, r_s_ = 0.011, p = 0.92). The consistency in boldness was also similar between individuals classed as more or less sociable, based on whether their mean time spent with a conspecific was more or less than, respectively, the population median value (figure S2B; quasipoission GLM: n = 80, F = 0.61, p = 0.44). The correlation in the time taken to leave the refuge over the two days to determine whether refuge use was consistent (figure S1A) was carried out for more and less sociable fish separately. There was no evidence that less sociable individuals were more repeatable in refuge use over repeated tests; in fact, more sociable fish showed a greater consistency in refuge use (figure S3A; Spearman’s rank correlation: n = 40, r_s_ = 0.47, p = 0.0024) than less sociable fish (figure S3B; n = 40, r_s_ = 0.31, p = 0.055). Body length did not correlate with the boldness consistency measure (Spearman’s rank: n = 80, r_s_ = 0.019, p = 0.87).

Supplementary Tables

Table S1: Full details of GLMMs presented in the main text and their statistical significance based on P values. With this frequentist approach in contrast to the AIC approach presented in the main text, the least significant main effect was removed from each GLMM and models rerun until only a single main effect remained. The LRT refers to the Likelihood Ratio Test, and SBL is standard body length. Significant effects at p < 0.05 are marked in bold. Sample sizes varied with analyses of different response variables as in some trials some response variables could not be recorded (for example, if neither fish left the refuge in paired fish tests, neither fish could be defined as the initiator and these trials would be excluded from the analysis of which fish left the refuge). The classification of ‘more’ or ‘less’ sociable individuals was performed for each response variable which ensured that the sample size for the more and less sociable tests were equal, and hence test power in each was determined only by the fitted coefficient and its variability. It did, however, allow a single individual with a mean sociability value close to the median to be classified as more sociable in some tests and less sociable in others. As in such a case, where the classification of more or less sociable is least accurate because they are closest to the threshold that defines the categories, we do not believe this is problematic as the differences between more and less sociable fish should be driven by those further from the threshold. Table S3 shows the results from the same models with this case removed, and the qualitative results remain unchanged, with the exception for the effect of boldness on whether an individual initiates into an arm of the maze, where the P value is just significant at 0.049 in the full data set and just non-significant at 0.055 with the case removed. In cases where an individual’s mean sociability was equal to the median mean sociability, the fish was not included in the analyses split by more or less sociable fish, thus the sample size of the analysis before splitting into more and less sociable fish is sometimes greater than the sum of the sample sizes in the tests where the data are split.

Table S2: Details of GLMMs including a sociability × boldness interaction term rather than splitting the data into less and more sociable fish. Boldness was log10 transformed before using as a covariate, as in the models presented in the main text, as was the sociability measure (note that in these models sociability is a covariate rather than individuals being categorised as less or more sociable). SBL is standard body length and the Null model is the model lacking any explanatory variables. d.f. refers to degrees of freedom and ΔAICc to the difference in the corrected Akaike Information Criterion between the model and the most likely model. Models are ordered within the analysis of each response variable by increasing ΔAICc. Also note that the models without sociability as a covariate are identical to those in tables 1 to 4 in the main text, and because the most likely model (ΔAICc = 0.0) in the table below for each response variable does not include sociability, the ΔAICc for each of the models not including sociability are identical to the corresponding values in tables 1 to 4.

In the analysis of whether an individual initiated leaving the refuge, there was little support for any model other than the one with boldness as the only explanatory variable, and thus little support for models including sociability. The model with the sociability × boldness interaction term was poorly supported (also see figure 2a and 2b, which shows that the slope of the effect of boldness is similar between less and more sociable fish). For the analysis of the time taken to leave the refuge by this initiating fish, again the most likely models included boldness as a main effect, and the model with the sociability × boldness interaction term was poorly supported. There was some support for the model with boldness and sociability as main effects, although the model with only boldness was the most likely and had fewer parameters. The most likely model for whether an individual initiated into an arm of the maze again only had boldness as a main effect, and the model with the sociability × boldness interaction term poorly supported. There was also support for the null model. In the analysis of the time taken to enter the arm by the initiating fish, the most likely model and models within 2 AICc units of the most likely model all included both boldness and body size. The well supported models, but not most likely model, also included sociability, both as a main effect only and as an interaction term with boldness. Other than this response variable, overall there was little support that sociability was important in directly affecting behaviour in the paired fish tests.

Table S3: Full details of GLMMs presented in the main text and their statistical significance based on P values, as in Table S1, with a single case removed where the fish was classified as more sociable in some tests and less sociable in others. See Table S1 for details.

Supplementary Figures


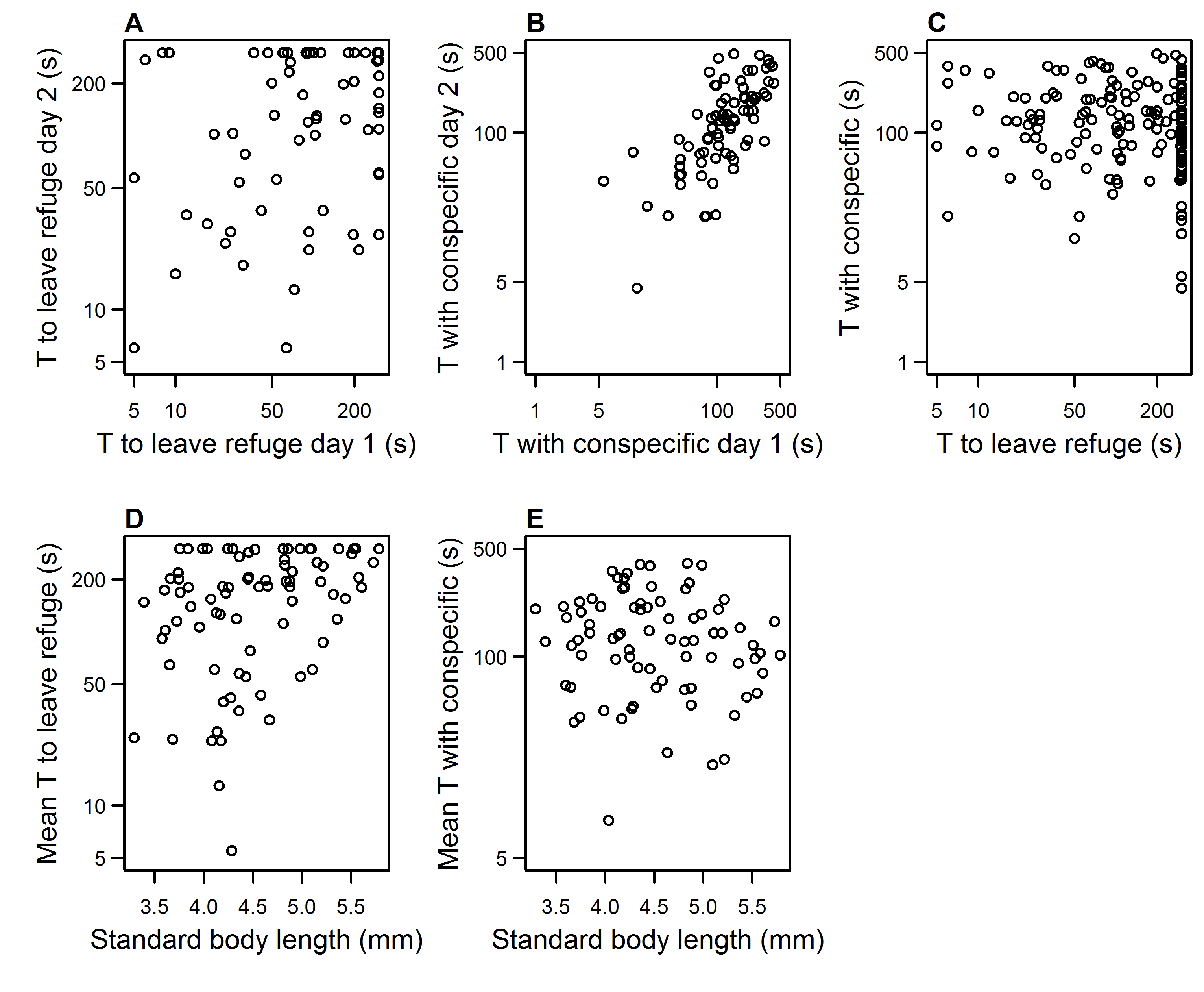


Figure S1. Correlations within and between boldness and sociability when fish were tested alone (i.e. single fish tests), and between boldness, sociability and standard body length (SBL). Boldness (A) was correlated between tests across the first and second days of testing, as was sociability (B). Boldness and sociability, correlated against each other within each day, were not correlated with one another (C). Bolder individuals have shorter latencies to leave the refuge, while more sociable individuals have longer times spent with the conspecific. Larger fish left the refuge later than shyer individuals (D), but body length did not correlate with the mean time spent with a conspecific (E).


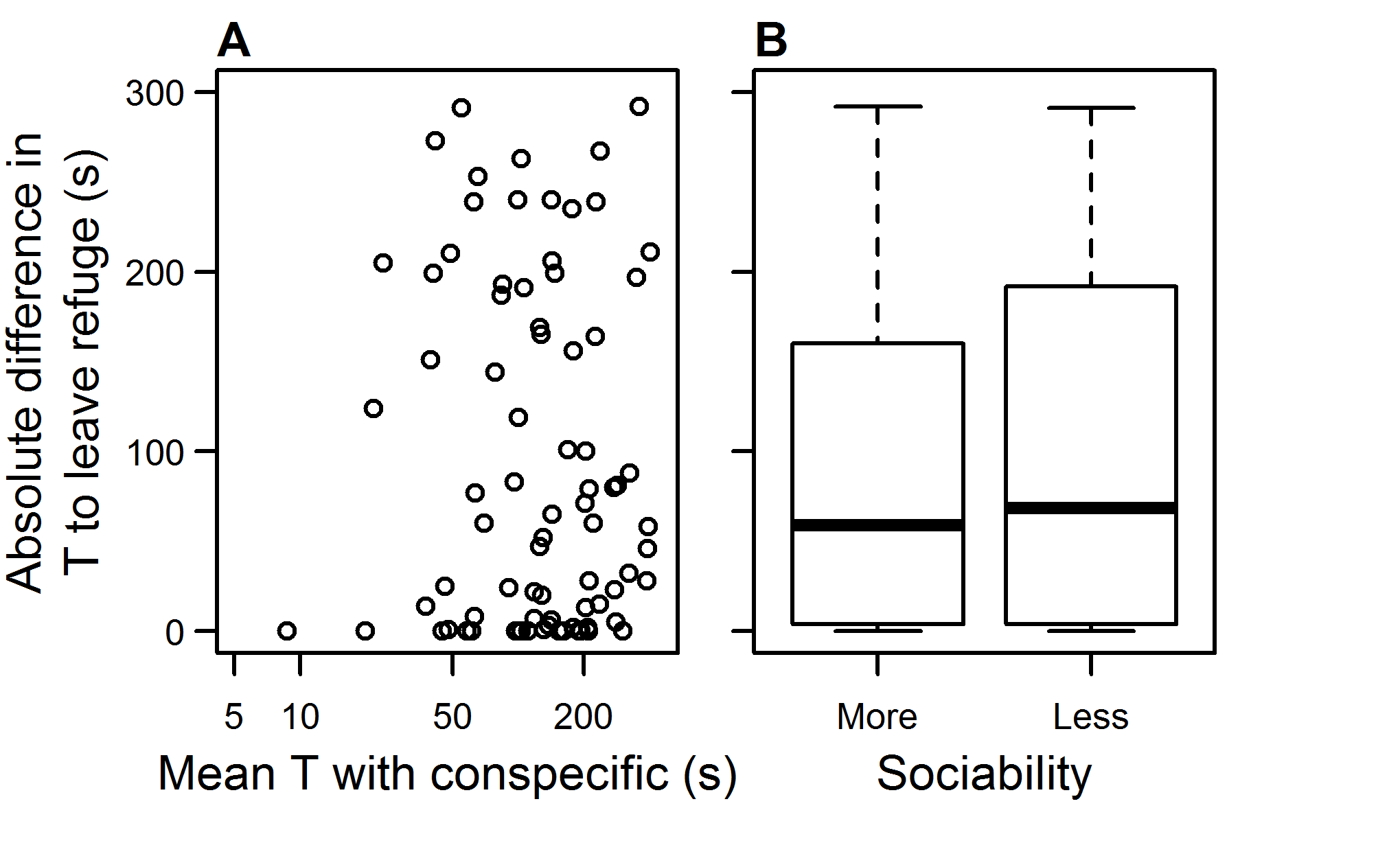


Figure S2. The relationship between individual social tendencies (sociability) and the change in latency to leave the refuge (boldness), i.e. consistency, between days 1 and 2 of single fish tests. Sociability (x axis in A) is plotted on a log10 scale. There was no correlation between sociability (mean time spent with a conspecific) and the absolute change in boldness (A), and correspondingly, there was no difference between more and less sociable fish (B).


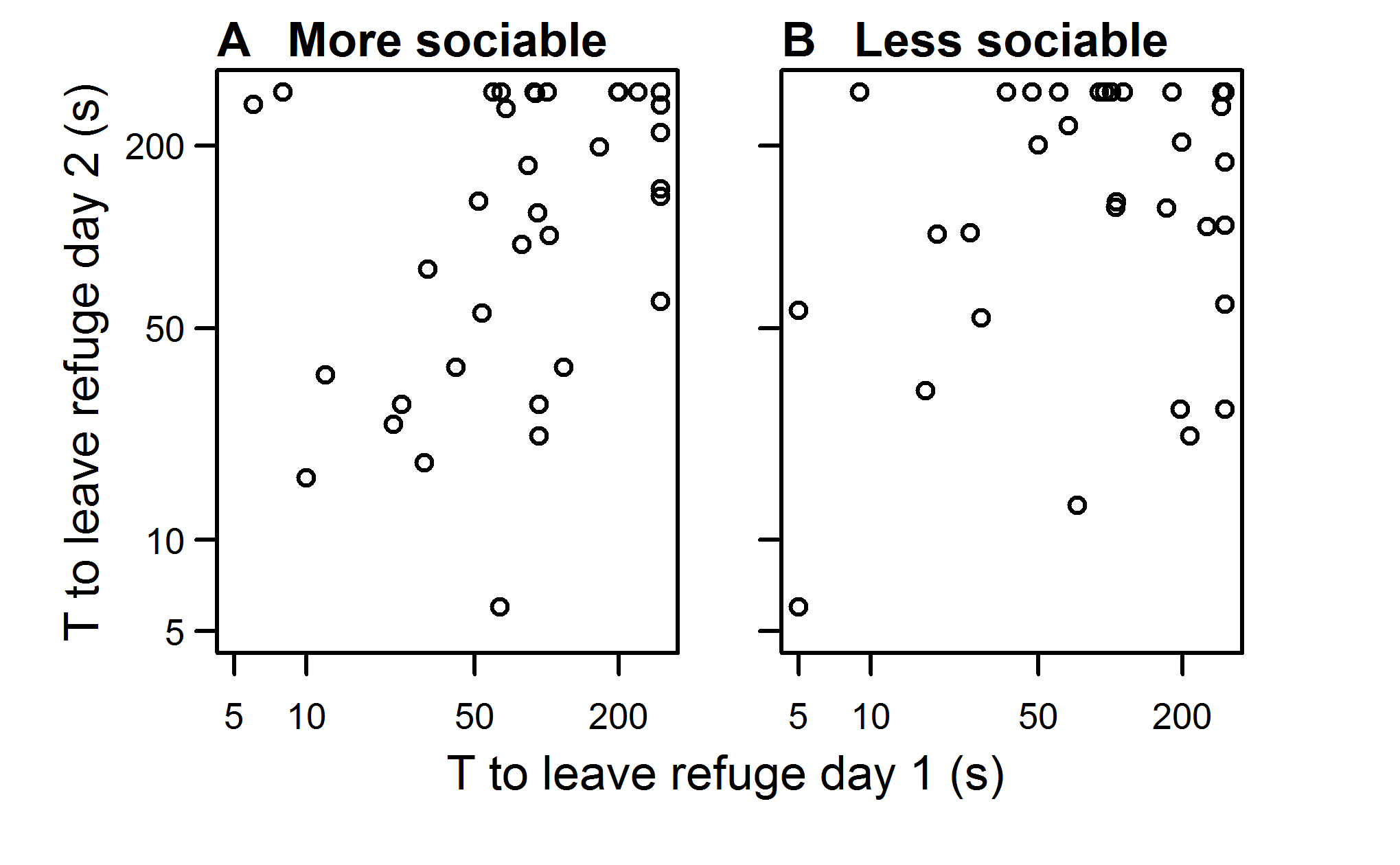


Figure S3. The correlation in latency to leave the refuge on days 1 and 2 for more (A) and less (B) sociable fish when fish were tested alone (i.e. single fish tests). Individuals with a mean time spent with a conspecific greater than the population’s median were classed as more sociable, and with a mean time spent less than the population’s median as less sociable. Note all scales are logged due to the skewed distributions of the latency to leave the refuge and time spent with a conspecific.

Data S1. Data from the experiment in .csv format. In the column names, S1 denotes data is from the first (day 1) single fish Y maze trial, S2 the second (day 2) single fish trial, P1 the first (day 1) paired fish test and P2 from the second (day 2) paired fish test. All times (“T”) are in seconds. SBL is the standard body length of each fish, in cm.
